# Heterogeneous Iron-Based Catalysts for Organic Transformation Reactions: A Brief Overview

**DOI:** 10.3390/molecules29133177

**Published:** 2024-07-03

**Authors:** Manash J. Baruah, Rupjyoti Dutta, Magdi E. A. Zaki, Kusum K. Bania

**Affiliations:** 1 Department of Chemistry, DCB Girls’ College, Jorhat 785001, Assam, India; manashl@tezu.ernet.in; 2 Department of Chemical Sciences, Tezpur University, Napaam, Tezpur 784028, Assam, India; 3 CSIR-North East Institute of Science and Technology, Jorhat 785006, Assam, India; rupjyotid@tezu.ernet.in; 4 Academy of Scientific and Innovative Research (AcSIR), Ghaziabad 201002, Uttar Pradesh, India; 5 Department of Chemistry, Imam Mohammad Ibn Saud Islamic University (IMSIU), Riyadh 11623, Saudi Arabia; mezaki@imamu.edu.sa

**Keywords:** heterogeneous, Fe catalyst, magnetic, organic reaction, sustainable chemistry

## Abstract

Iron (Fe) is considered to be one of the most significant elements due to its wide applications. Recent years have witnessed a burgeoning interest in Fe catalysis as a sustainable and cost-effective alternative to noble metal catalysis in organic synthesis. The abundance and low toxicity of Fe, coupled with its competitive reactivity and selectivity, underscore its appeal for sustainable synthesis. A lot of catalytic reactions have been performed using heterogeneous catalysts of Fe oxide hybridized with support systems like aluminosilicates, clays, carbonized materials, metal oxides or polymeric matrices. This review provides a comprehensive overview of the latest advancements in Fe-catalyzed organic transformation reactions. Highlighted areas include cross-coupling reactions, C–H activation, asymmetric catalysis, and cascade processes, showcasing the versatility of Fe across a spectrum of synthetic methodologies. Emphasis is placed on mechanistic insights, elucidating the underlying principles governing iron-catalyzed reactions. Challenges and opportunities in the field are discussed, providing a roadmap for future research endeavors. Overall, this review illuminates the transformative potential of Fe catalysis in driving innovation and sustainability in organic chemistry, with implications for drug discovery, materials science, and beyond.

## 1. Introduction

The synthesis of organic compounds lies at the heart of chemistry, serving as the foundation for advancements in medicine, materials science, and numerous other fields [1,2]. Over the years, scientists have developed a myriad of synthetic methods to construct complex molecules with precision and efficiency [3,4]. Among these methods, catalysis stands out as a powerful strategy, allowing for the selective activation of chemical bonds and the facilitation of otherwise challenging transformations [5,6]. Transition metal catalysis has emerged as a cornerstone of modern synthetic chemistry, enabling a broad range of transformations that were once thought to be impossible or impractical [6,7]. Metals such as palladium (Pd), platinum (Pt), gold (Au), and ruthenium (Ru) have traditionally dominated this field, owing to their ability to catalyze a wide array of reactions with high efficiency and selectivity [6,7,8,9,10]. However, the scarcity and high cost of these noble metals have spurred efforts to develop catalytic systems based on more abundant and inexpensive alternatives.

In recent years, iron (Fe) has emerged as a particularly promising candidate for catalysis, owing to its abundance, low cost, and low toxicity [11,12]. Once considered a stoichiometric reagent or a catalyst for relatively simple reactions, Fe has undergone a renaissance in catalysis, fueled by advances in heterogeneous catalysis, owing to its several advantages [13,14]. The resurgence of heterogeneous Fe catalysis can be attributed to several key factors. Firstly, the desire to reduce the environmental impact of synthetic chemistry has prompted researchers to seek alternatives to precious metal catalysts [12]. Fe, as an Earth-abundant element with low toxicity, represents an attractive candidate for addressing these concerns [11,15]. Additionally, advances in catalyst design and reaction engineering have led to the development of heterogeneous Fe catalysts with improved activity, selectivity, and stability, enabling the synthesis of a wide range of organic compounds under mild conditions [16]. This potential has led to a wave of chemical research focused on harnessing the catalytic potential of Fe for a diverse array of organic transformations [12,14].

**Scheme 1:**
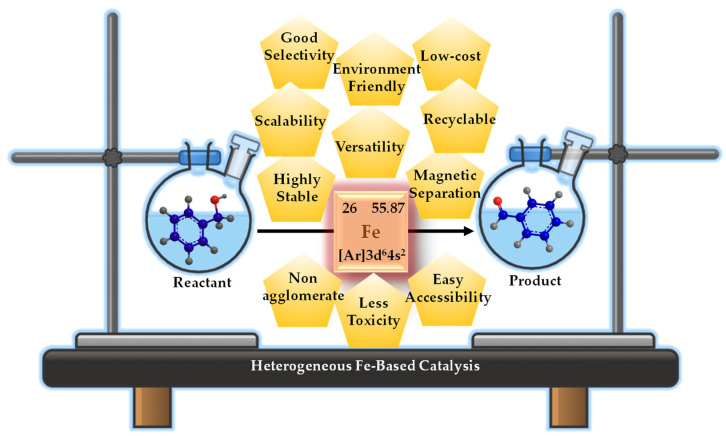
Advantages of heterogeneous Fe-based catalysts in organic transformation reactions.

Heterogeneous Fe-based catalysts offer several advantages: Scheme 1 [16,17]. Apart from the cost-effective and environmentally friendly nature, they can be easily separated from reaction mixtures, facilitating catalyst recovery and reuse [16]. These catalysts typically exhibit high stability and durability under reaction conditions. They exhibit high thermal and chemical stability, making them suitable for a wide range of reaction conditions [18]. These catalysts often simplify purification processes by reducing product contamination with catalyst residues. Additionally, heterogeneous Fe catalysts can be engineered for high selectivity in specific reactions [14]. Their robustness and ease of handling make them advantageous for large-scale industrial processes [13]. Capable of catalyzing a broad spectrum of organic transformation reactions, these catalysts also limit side reactions within their confined environment, thereby improving overall yield and efficiency [17]. Moreover, magnetic nanoparticles (NPs) offer significant potential in heterogeneous catalysis due to their easy separation and reusability [19]. Their saturation magnetization depends on size and surface properties, with small nanoparticles prone to aggregation [20]. Coating them with materials like silica, carbon, metals, metal oxides, or polymers prevents aggregation and enhances catalytic activity by supporting active species on their high surface area [21]. The field of heterogeneous Fe-catalyzed organic transformation reactions has been expanding rapidly in the last several years [16,17]. A broad range of organic reactions, including hydrogenation, oxidation, activation, coupling, and cycloaddition processes, have been described in the literature [16,17]. These reactions involve a wide range of bond formations, including aryl–aryl, aryl–alkyl, alkyl–alkyl, and heteroatom cross-couplings, among others [16,17]. Moreover, heterogeneous Fe catalysts have been shown to exhibit high functional group tolerance, allowing for the direct coupling of substrates bearing a variety of functional groups without the need for pre-activation or protection strategies [22]. One of the most significant areas of progress in heterogeneous Fe catalysis lies in cross-coupling reactions, a class of transformations that are widely used in organic synthesis for the formation of carbon–carbon and carbon–heteroatom bonds [17,22]. Usually, cross-couplings have been dominated by Pd and other precious metals, which offer high reactivity and selectivity but are limited by their scarcity and high cost [23]. In recent years, however, heterogeneous Fe catalysts have emerged as viable alternatives for cross-coupling reactions, offering competitive reactivity and selectivity profiles while addressing the limitations of precious metal catalysts [22]. In addition to cross-coupling reactions, heterogeneous Fe catalysis has found applications in a variety of other synthetic transformations. For example, Fe-catalyzed C–H activation and functionalization reactions have emerged as powerful tools for the direct functionalization of C–H bonds, enabling the synthesis of complex molecules with high efficiency and selectivity [24]. Similarly, heterogeneous Fe catalysts have been employed in asymmetric transformations, providing access to enantioenriched compounds with high levels of stereo control [25]. Additionally, Fe catalysts have been used in cascade reactions, in which multiple bond-forming events occur sequentially in a single reaction vessel, leading to the rapid synthesis of complex molecular architectures [26]. Recent advancements highlight the diverse applications of magnetic nanomaterials in catalysis, including hydrogenation, Suzuki–Miyaura coupling, oxidation, chiral catalysis, enzyme catalysis, photocatalysis, electrocatalysis, and photoelectrochemical catalysis [17,27].

**Scheme 2:**
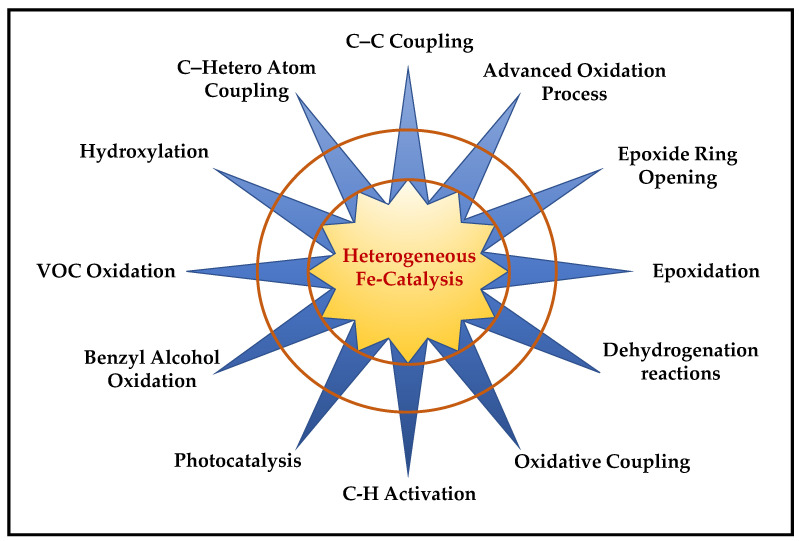
Perspectives of Fe-catalyzed organic reactions.

In this article, we will provide a comprehensive overview of the recent advances in heterogeneous Fe-catalyzed organic transformation reactions. A brief exploration will be performed in this article within the time period 2019–2024, including the application of heterogeneous Fe catalysis in a variety of synthetic transformations, together with cross-coupling reactions, C–H activation, coupling reactions, oxidation, hydroxylation reactions, etc. (Scheme 2). Finally, we will discuss the current challenges and future directions in the field, highlighting opportunities for further innovation and development. Through this brief review, we expect to provide insights into the transformative potential of heterogeneous Fe catalysis and inspire further research in this exciting and rapidly evolving area of chemistry.

## 2. C–H Activation Reaction

C–H activation is a chemical process where a C–H bond within an organic molecule is cleaved or ‘activated’ to form new bonds, typically involving the introduction of functional groups [28,29]. This reaction is significant in synthetic chemistry as it allows for the direct transformation of simple and abundant hydrocarbons into more complex and valuable molecules without the need for pre-functionalization [30,31]. C–H activation reactions often require transition metal catalysts to facilitate the bond cleavage and subsequent functionalization steps [32]. These reactions have broad applications in the synthesis of pharmaceuticals, agrochemicals, materials, and fine chemicals, offering efficient routes to structurally diverse compounds [33,34,35]. Research shows that a good number of heterogeneous Fe catalysts have been employed in various C–H activation reactions, including cross-coupling, cyclization, and functionalization reactions, contributing significantly to the development of sustainable synthetic methodologies [36,37,38]. E. Nakamura is regarded as a pioneer in the field of C–H activation reactions. He and his research group have previously shown a comprehensive analysis of previous studies on C–H activation processes catalyzed by Fe [39]. His research showcased the activation of C–H bonds and subsequent reactions with alkynes to form cyclized products [40,41]. Nakamura’s achievements include the direct phenylation of isoxazoles using palladium and the methylation of naphthylamide with iron catalysts [42]. These works have broadened the scope of C–H activation, emphasizing the use of economical and less toxic metals like iron in these chemical processes [43]. However, it is noteworthy to mention herein that Lutz Ackermann is a well-known chemist who has made significant contributions to the field of C–H activation [44,45,46]. Ackermann’s work in this area has focused on developing new catalysts and methodologies to facilitate these transformations with greater efficiency and selectivity [47,48,49]. In addition to Ackermann’s contributions, Professor G. Cera and S. Cattani have also reviewed recent advancements in iron-catalyzed C–H activation reactions, particularly focusing on synthetic chemistry applications [50]. Very recently, Lemmens et al. derived an Fe-based metal–organic framework (MOF), CAU-27-Fe as a suitable heterogeneous catalyst for C–H activation reaction [33]. The CAU-27-Fe was utilized for direct C–H amination of ether by N-heterocycles under mild reaction conditions. This highly stable catalyst was reported to be reused for multiple runs, making it an efficient and sustainable option for novel C–H activation processes. The discussed protocol is particularly suitable for the synthesis of key pharmaceutical precursors, enhancing its relevance in the field of drug development. Zhang et al. developed a photo-electrocatalytic methodology for C–H amination of arene using robust and earth-abundant hematite (α-Fe_2_O_3_) as the catalyst [51]. This technique enabled the preparation of various heterocycles with aryl C–N moieties from simple arenes and azoles without looking for pre-functionalization. The reported approach could be successfully applied to the functionalization of several pharmaceutical molecules, pioneering the use of photoelectrochemical cells in organic synthesis. Recently, Devaranjan and Suresh developed an Fe-based sustainable heterogeneous MOF catalyst, Fe(BTC), which was proven to be highly effective for synthesizing 2,4-diarylquinolines [52]. This reported method avoided the need for stoichiometric amounts of catalyst and sensitive reagents, producing 2,4-diarylquinolines in good to excellent yields with broad functional group tolerance. This MOF was found to be easily separated by filtration and reused over five times without significant loss of activity. In another work, To et al. synthesized one more Fe-MOF (VNU-21) using 1,3,5-benzenetricarboxylic acid (BTC), 4,4′-ethynylenedibenzoic acid, and FeCl2 for the one-pot synthesis of quinazolinones [53]. In this procedure, Fe-catalyzed oxidative Csp3–H bond activation was used to decarboxylate phenylacetic acids, followed by the metal-free oxidative cyclization using 2-aminobenzamides. Additionally, the catalyst was found to be recycled up to a good number of catalytic cycles without experiencing a discernible decrease in performance. Again, a new Fe-MOF, VNU-22 {[Fe_3_(BTC)(BPDC)_2_]·11.97H_2_O}, was synthesized by Doan et al., using BTC^3–^ (1,3,5-benzenetricarboxylate), BPDC^2–^ (4,4′-biphenyldicarboxylate), and an Fe–carbonyl complex [Fe_3_(CO_2_)_7_] [54]. The VNU-22 was reported to efficiently catalyzed the synthesis of 2,4,6-triphenylpyridines from acetophenones, phenylacetic acids, and ammonium acetate through the C–H activation process. The MOF was easily recovered and reused without significant loss of catalytic performance, representing an industrially attractive synthetic pathway.

**Scheme 3:**
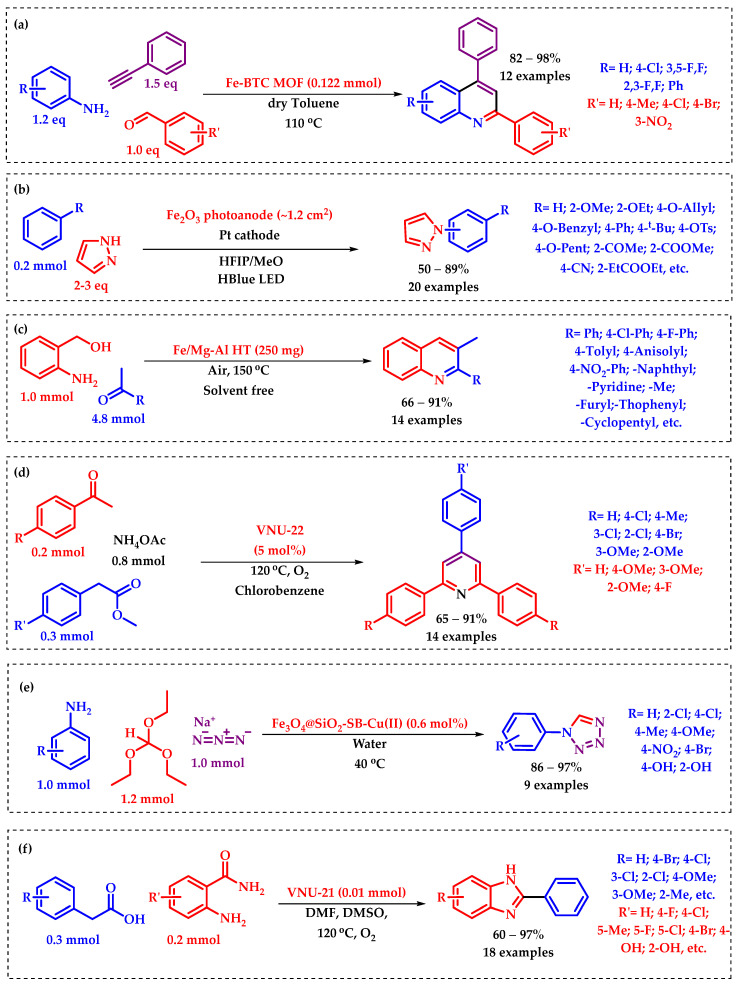
(**a**) [52], (**b**) [51], (**c**) [55], (**d**) [54], (**e**) [56], and (**f**) [53]. Some significant reported outcomes of heterogeneous Fe-catalyzed C–H activation reactions.

Recently, Waghchaure et al. derived a superior heterogeneous catalyst of Fe doped ZnO (Fe-ZnO) for Biginelli reaction for the production of 3,4-dihydropyrimidin-2-one derivatives [57]. Using a one-pot, three-component reaction with urea, a β-dicarbonyl compound, and various aromatic aldehydes, a minimal amount of the Fe-ZnO catalyst was reported to be efficiently synthesized 3,4-dihydropyrimidinones. Furthermore, this protocol offered advantages such as short reaction time, high product purity, catalyst recyclability and reusability. A bifunctional heterogeneous catalyst, based on Mg-Al hydrotalcite, was designed by Motokura et al. in porous FeO(OH) to facilitate the one-pot synthesis of 2-substituted quinoline derivatives via dehydrogenative oxidation-cyclization processes [55]. Without the need for extra homogenous bases or solvents, this reported protocol proceeded well in an open atmosphere under cost-effective Fe catalysis. Julián E. Sánchez-Velandia and Aída Luz Villa designed Fe(III)-based heterogeneous catalysts supported on MCM-41 for α- and β-pinene epoxide isomerization [58]. The synthesized catalyst exhibited excellent productivity for the α-pinene epoxide isomerization reaction with a high turnover number (TON) of 364 along with greater % conversion (73%) and selectivity (59%) towards the formation of campholenic aldehyde. For β-pinene epoxide, the catalyst had the highest TON of 299 with a 60% conversion and 100% selectivity to myrtanal. The activity of the catalyst was also correlated with metal oxides, particle diameter, as well as acidity strength. However, the catalyst maintained its heterogeneity over five cycles with moderate to good activity. A new environmentally friendly Silica coated (SiO_2_) Fe-oxide-based heterogeneous catalyst, Fe_3_O_4_@SiO_2_-Im(Br)-SB-Cu(II), was synthesized by Mashhoori and Sandaroos, and utilized for generating tetrazole derivatives in water media [56]. The reported catalyst showed exceptional efficiency with 97% yield of tetrazole. The catalyst’s efficacy was also studied and was credited to the combined action of the Fe-center and the imidazolium ion. Additionally, it could be reused for several catalytic cycles without significant loss in catalytic activity and without causing contamination. Scheme 3 demonstrates some interesting heterogeneous Fe-catalyzed C–H activation processes. 

## 3. C–C Coupling Reactions

**Scheme 4:**
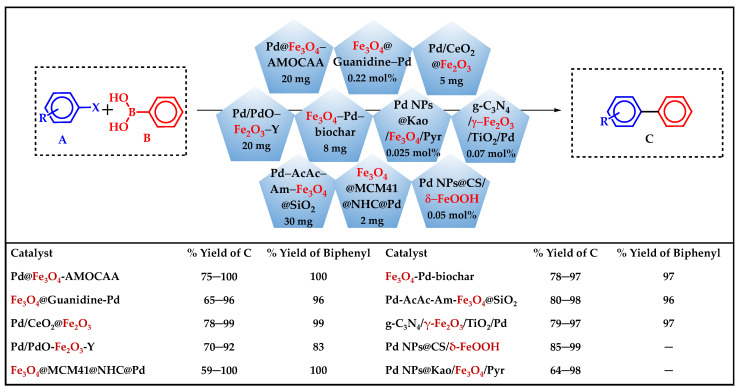
Recently reported Fe-oxide based magnetic heterogeneous catalysts for SM coupling reactions.

C–C coupling reactions catalyzed by heterogeneous Fe represent a transformative approach in organic synthesis, harnessing the abundance, low cost, and eco-friendliness of Fe as a transition metal catalyst [59,60]. These reactions involve the formation of C–C bonds between two or more organic substrates, enabling the construction of complex molecular architectures with high efficiency and selectivity [60]. The field of C–C coupling reactions has witnessed remarkable advancements with Fe catalysts, showcasing their versatility across a spectrum of synthetic methodologies [59]. From traditional cross-coupling reactions to novel transformations like direct C–H functionalization, Fe catalysis has emerged as a powerful tool for chemists seeking sustainable and economical routes to intricate molecular structures [59,60]. Moreover, the development of ligands and reaction conditions tailored to Fe catalysis has expanded the scope of these transformations, allowing access to previously inaccessible chemical space [61]. In addition to fundamental advancements, recent years have witnessed the application of Fe-catalyzed C–C coupling reactions in the synthesis of complex natural products, pharmaceuticals, and functional materials [28]. These transformative achievements highlight the practical significance of Fe catalysis in addressing societal needs and advancing drug discovery, materials science, and sustainable chemistry. Recently, Jang et al. conducted an in-depth analysis on hybrid Pd–Fe_3_O_4_ nanocatalysts, with specific attention given to urchin-like FePd–Fe_3_O_4_, Pd/Fe_3_O_4_, Pd/Fe_3_O_4_/charcoal, as well as flower-like Pd–Fe_3_O_4_ nanocomposites [62]. These nanocomposites are utilized as effective catalysts for various C–C coupling reactions, such as Suzuki-Miyaura (SM), Heck, and Sonogashira reactions, showcasing superior catalytic activity and reusability compared to many previously reported catalysts, owing to their magnetic properties. Due to their well-organized morphology and magnetic characteristics these hybrid Fe-based nanostructures give outstanding performance in terms of product yield, stability of the catalyst, and recyclability. Similarly, Hegde et al. designed a Pd functionalized nanocatalyst using silica-coated Fe-oxide and furaldehyde (FA) Schiff base as a supported material (Fe_3_O_4_@SiO_2_–FA–Pd) [63]. Using the synthesized Fe_3_O_4_@SiO_2_-FA-Pd NPs as a magnetic nanocatalyst, the Mizoroki–Heck reaction of arylbromide and terminal alkenes resulted in effective reactivity with excellent yields. Very recently, Sonawane et al. developed a silica-supported Fe-oxide-based catalyst, Pd-SILP-Fe_3_O_4_@SiO_2_, for Sonogashira coupling in aqueous media under aerobic conditions [64]. The synthesized nanocatalyst demonstrated excellent thermal stability, high catalytic efficiency, elevated turnover frequencies (TOFs), simple magnetic recovery, high recyclability, compatibility with aqueous systems, and thereby facilitating the reactions using water as a green solvent. Sardarian et al. developed a new Fe-oxide-based organometallic catalyst by binding Pd(0) to polyvinyl alcohol-modified Fe_3_O_4_@SiO_2_ nanospheres, designated as Fe_3_O_4_@SiO_2_-PVA-Pd(0) [65]. The system employed in Heck and Sonogashira coupling reactions in aqueous environments. Interestingly, it demonstrated outstanding performance with minimal Pd content, yielding excellent product yield and attaining high TOFs. Additionally, the catalysts behaved as magnetically retrievable with high recyclability rates. Again, a biochar-based nanocomposite catalyst was developed by Akay et al. by sequentially depositing Fe_3_O_4_ and Pd onto a biochar surface derived from waste biomass [66]. The Pd–Fe_3_O_4_ biochar catalyst exhibited high catalytic efficiency for the SM coupling reaction across various experimental conditions, bases, and aryl halides. It achieved an optimal yield of 99% in the coupling reaction between aryl iodide and phenylboronic acid using only 8 mg of catalyst, K_2_CO_3_ as the base, and in a solvent-free setup with microwave irradiation. The catalyst’s broader applicability to SM coupling reactions was demonstrated with a variety of aryl chlorides, bromides, and iodides. Moreover, the catalyst maintained consistent activity over eight consecutive runs, underscoring its potential for recovery and reuse. To perform SM and Sonogashira coupling reactions, Tamoradi et al. designed a bifunctional reusable Fe-oxide-based catalyst (Pd@Fe_3_O_4_/AMOCAA) by complexing Pd with magnetic Fe_3_O_4_ nanoparticles coated with a 2-(7-amino-4-methyl2-oxo-2H-chromen-3-yl)acetic acid (AMOCAA) [67]. The advantages of the system include the use of non-toxic, commercially available, or easily accessible starting materials for the synthesis of the catalyst, straightforward recovery, high product yields in short reaction times, operational simplicity, robust activity, and stability, making it highly suitable for industrial and pharmaceutical applications. Similarly, Halligudra et al. introduced an Fe_3_O_4_@Guanidine-Pd nanocatalyst effective for SM cross-coupling of aryl halides with phenylboronic acids and for selectively reducing nitroarenes to their corresponding amines [68]. The prepared Fe_3_O_4_@Guanidine-Pd demonstrated efficient catalytic performance by achieving conversion of aryl halides to biaryl derivatives in aqueous medium with minimal catalyst (0.22 mol %) and shorter reaction time while maintaining high TON and TOFs. Notably, the synthesized Fe_3_O_4_@Guanidine-Pd was reported to be magnetically separable, with a very high recyclability rate without significant loss of catalytic activity. Recently, Bora et al. designed a novel nanocatalyst consisting of Pd/PdO and Fe_2_O_3_ NPs, zeolite-Y support, and Pd/PdO-Fe_2_O_3_-Y [69]. The catalyst was reported to be effective for C–Cl bond activation in aryl chlorides for SM cross-coupling, achieving high yields (up to 92%) with very low Pd loading (0.0037 mol %). The catalyst exhibited excellent thermal stability, and high recyclability. The outstanding productivity of the synthesized nanocatalyst was attributed to the participation of surface hydroxyl groups and modification of basic sites in zeolite-Y. Akkoc et al. developed an Fe_3_O_4_@MCM-41@NHC@Pd catalyst, formed by attaching Pd metal to the N-heterocyclic carbene (NHC) ligand on Fe_3_O_4_@MCM-41, which showed excellent catalytic activity in SM reactions [70]. With just 2 mg of catalyst, it achieved high TOF (up to 408,404 h^−1^) at room temperature in 2-propanol/H_2_O (1:2) solvent media. Similar to the above cite reports, the catalyst could be easily recovered and reused multiple times without notable performance loss, indicating excellent structural and chemical stability. Çalışkan et al. synthesized an Fe-oxide (δ-FeOOH)-based Pd nanocatalyst (Pd NPs@CS/δ-FeOOH) attached to chitosan and utilized this in synthesizing biaryls via an SM reaction with satisfactory yields [71]. Moreover, the catalyst maintained its activity over eight cycles, indicating its potential for use in diverse organic reactions due to its promising performance and reusability. Recently, Sheikh et al. developed a recyclable magnetic nano Pd-complex, having Fe_2_O_3_ NPs, which exhibited high efficiency as a catalyst for SM and Mizoroki–Heck coupling reactions [72]. Remarkably, the catalyst displayed high activity in water at moderate reaction conditions (90 °C), with a reasonable reusability rate. Vibhute et al. designed an Fe-oxide-based novel nanomagnetic catalyst (Pd-AcAc-Am-Fe_3_O_4_@SiO_2_) containing Pd over SiO_2_ support [73]. The system demonstrated notable efficacy in catalyzing SM cross-coupling reactions of aryl halides with phenylboronic acid. Its performance was evaluated under different conditions, showing excellent yields, mild reaction conditions, short reaction times, and easy magnetic work-up. It exhibited recyclability over at least six cycles without significant loss of activity, making it a promising candidate for sustainable catalysis. A new Fe-based heterogeneous catalyst (Pd NPs@Kao/Fe_3_O_4_/Pyr) was established by Baran et al. by anchoring Pd NPs onto Fe_3_O_4_-loaded Schiff-base-modified kaolin [74]. The nanostructure was found to be suitable for SM cross-coupling reactions with high activity across various aryl halides with different functional groups. Additionally, it displayed excellent recyclability, maintaining an 89% yield even after 10 consecutive runs. On the other hand, the photocatalyzed C–C coupling process has been a thriving area of research and development in organic synthesis chemistry and photocatalysis in recent years, offering a promising new approach to C–C bond fabrication. Giving importance to light energy, Liu et al. designed Pd/CeO_2_@Fe_2_O_3_ heterojunctions as an efficient photocatalyst for an SM reaction using solar energy at room temperature [75]. This structure of the material was reported to be layered double hydroxide (LDH), which facilitated light absorption and exposed more active sites (Pd). The resulting abundant oxygen vacancies and Pd/CeO_2_@Fe_2_O_3_ heterojunctions extended the response region of visible light and aided in electron–hole pair separation. The directional transfer of electrons from Fe to Pd accelerated oxidative addition in the SM reactions, leading to high catalytic activity. The catalyst achieved a TOF of 1770 h^−1^ under visible light. Jahanshahi et al. synthesized an Fe-based magnetic nanocomposite, g-C_3_N_4_/γ-Fe_2_O_3_/TiO_2_/Pd, synthesized as a visible-light photocatalyst, efficiently catalyzed Hiyama and SM cross-coupling reactions, including some challenging aryl chlorides [76]. The catalyst was found to be operative effectively at room temperature under visible light irradiation. As usual, the material could be magnetically separated and reused for up to seven cycles without significant loss of activity. Adam et al. designed Fe_3_O_4_-supported Cu(II) catalysts immobilized on TiO_2_-coated Fe_3_O_4_ nanoparticles (CuL@TiO_2_@Fe_3_O_4_, where L is tridentate iminoisonicotine ligand) [77]. The catalyst was tested in SM coupling and Buchwald–Hartwig (BH) cross-coupling reactions and had an excellent catalytic performance. The reusability test of the catalyst exhibited very good outcomes with high reusability rates. From these scientific analyses, we noticed that magnetic Fe-based materials present a promising additive to Pd catalysts in SM coupling reactions, offering advantages in terms of cost, environmental impact, and ease of recovery (Scheme 4). Continued research into optimizing these systems and understanding their mechanisms will further enhance their applicability and efficiency in organic synthesis.

**Scheme 5:**
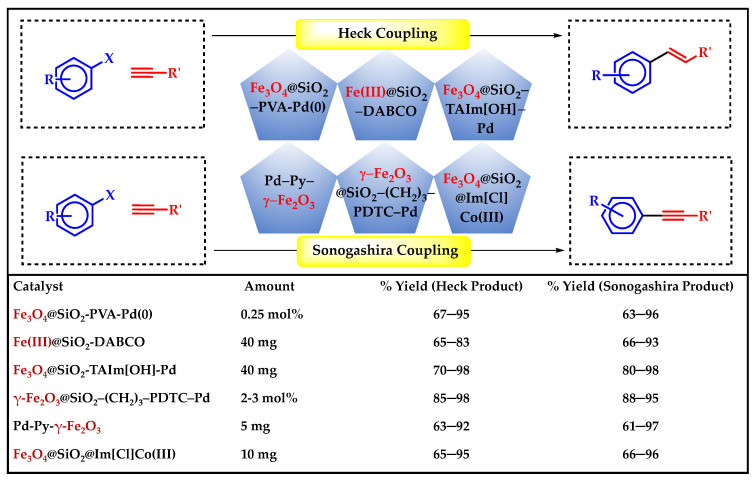
Some recently reported Fe-oxide based bifunctional catalysts for Heck and Sonogashira coupling reactions [65,79,80,81,82,83].

Nevertheless, while most reports designate Pd as the primary metal for such coupling reactions, there are also some reports discussing Pd-free Heck coupling reactions. Deepa et al. established a chloroglycine–ionic liquid imidazolium supported Fe^3+^ complex as an effective and green catalyst for the Heck reaction [78]. The remarkable features of this catalyst system include the achievement of excellent yields of trans-stilbenes (up to 94%) with just 0.005 mol % of catalyst, and the ability for the catalyst to be readily recovered and reused without significant loss of activity. Similarly, Hajipour et al. reported an Fe-catalyst, Fe(III)@SiO_2_-DABCO, for the cross-coupling of aryl iodides, providing a mild basic environment (K_2_CO_3_ as the base) [79]. The system enables Heck reactions under mild conditions (Pd-free, H_2_O solvent, 80 °C, short reaction time). The iron catalyst is highly active and recyclable by simple filtration, offering an economical approach. Its use instead of palladium or copper enhances environmental, economic, and industrial benefits. Additionally, the system facilitates Fe-catalyzed Sonogashira and Heck reactions, expanding its utility in organic syntheses. Similarly, Min et al. developed a versatile and recyclable magnetite and NHC ligand-based catalyst, Fe_3_O_4_@SiO_2_-TAIm[OH]-Pd for the efficient Heck, Suzuki, and Sonogashira cross-coupling reactions under mild conditions [80]. The NHC ligand precursor was immobilized on magnetite, and its catalytic activity was assessed in these coupling reactions as a heterogeneous catalyst. Again, Sobhani et al. synthesized a novel immobilized Pd-pyridine (Py) complex on γ-Fe_2_O_3_ magnetic nanoparticles, i.e., Pd-Py-γ-Fe_2_O_3_ [81]. The catalytic activity of this synthesized catalyst was examined in Heck, Suzuki, and Sonogashira coupling reactions with various aryl halides. The catalyst could be easily separated from the reaction mixture using an external magnetic field and reused multiple times without significant loss of catalytic activity. Another SiO_2_ supported Fe-oxide-based catalyst was also reported by Tashrifi et al., and involved the preparation of a Pd complex of (pyridin-2-ylmethyl)dithiocarbamate (PDTC) supported on γ-Fe_2_O_3_@SiO_2_ nanoparticles, i.e., γ-Fe_2_O_3_@SiO_2_–(CH_2_)_3_–PDTC–Pd [82]. The catalytic performance of this nanomaterial was assessed in Heck and Sonogashira coupling reactions between various aryl halides and different alkenes or phenylacetylene in water. The synthesized nanocatalyst demonstrated high catalytic activity even in very small quantities (<0.1 mol %), and could be reused for ten successive catalytic cycles without any significant activity loss. Similar to the above report, another Fe-oxide-based magnetic catalytic system has been developed by Kazemnejadi et al. for Heck and Sonogashira cross-coupling reactions by decorating the Schiff base Co(III) complex on Fe_3_O_4_@SiO_2_ nanoparticles, designated as Fe_3_O_4_@SiO_2_@Im[Cl]Co(III)-melamine nanocomposite) [83]. The catalyst demonstrated compatibility with a variety of substrates, achieving high to excellent yields for both Heck and Sonogashira coupling products. Additionally, the catalyst proved its true heterogeneity by empowering its recyclability rate up to the seventh consecutive run. The reactions were conducted using ethanol as the solvent and under base-free conditions, making them suitable from a green and sustainable chemistry perspective. Scheme 5 depicts some of the recently reported Fe-based interesting bifunctional catalytic systems for Heck and Sonogashira coupling reactions.

## 4. C–N Bond Formation Reactions

**Scheme 6:**
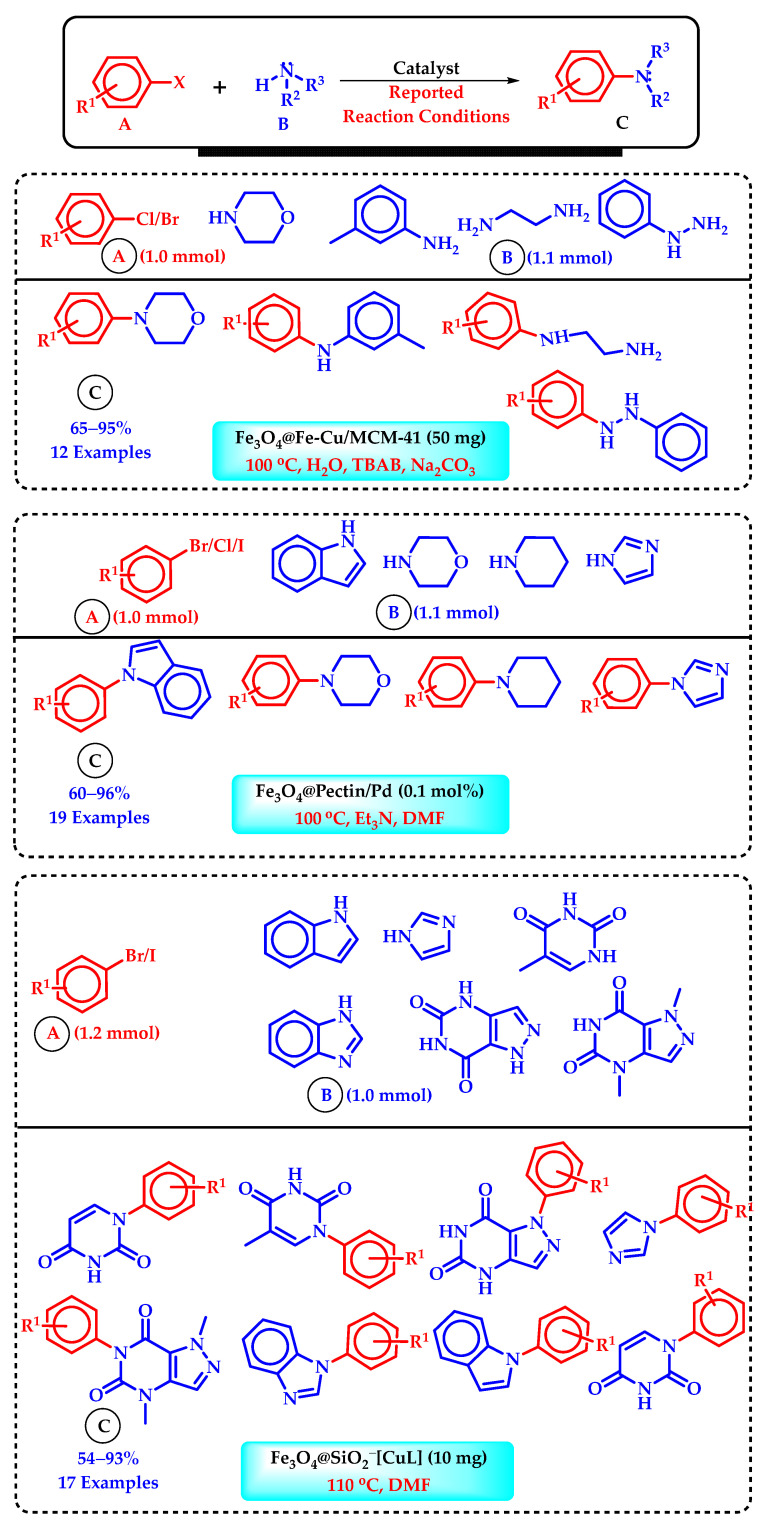
Representation of some significant Buchwald–Hartwig amination products synthesized using recently reported heterogeneous Fe catalysts [88,90,92].

The presence of carbon–heteroatom (basically O, N, S) bonds is common in many natural products, medicines, and insecticides [84]. The creation of carbon–heteroatom bonds has, therefore, received a lot of focus for the conception of easy and efficient procedures. Transition-metal-catalyzed cross-coupling reactions represent a promising area of modern organic synthesis, offering efficient and sustainable methods for the construction of carbon–heteroatom bonds [85]. Due to its uniqueness, iron catalysis has consistently been a favored option for such reactions. [12]. The Fe catalyst, a first-row transition metal, typically facilitates cross-coupling reactions by means of a single-electron transfer (SET) process. There are only a handful of reported cases where a two-electron transfer process was involved, demonstrating a distinct reactivity with second- and third-row transition metals, which consistently boost cross-coupling reactions through means of a two-electron transfer process [86]. The production of carbon–heteroatom bonds has lagged behind that of carbon–carbon bonds, despite the abundance of research on carbon–carbon bonding via Fe catalysis [12]. The transition-metal-catalyzed formation of C-N bonds stands out as a pivotal process in organic synthesis, finding widespread application across chemical, pharmaceutical, and materials industries [87]. Traditionally, the BH amination reaction has been the go-to method for accessing C–N bonds, involving the cross-coupling of aryl or alkenyl halides with amines. However, recent years have seen significant advancements in the direct formation of C–N bonds via C–H activation [88]. Despite substantial progress achieved with transition metals such as Cu, Pd, Ni, Fe, and Co, Fe-catalyzed C–N bond formation has received relatively less attention, possibly due to concerns regarding the adverse effects of trace metals. Going through the most recent literature, it was noticed that a lot of research groups presented a good number of results regarding BH amination reactions (Scheme 6). Chahkamali et al. demonstrated an Fe_2_O_3_-supported Pd–N-heterocyclic carbene complex for fluoride-free Hiyama, SM, and cyanation (C–N coupling) reactions in pure water [89]. This catalyst effectively yielded various biaryls and aryl nitriles from aryl halides with triethoxyphenylsilane, phenylboronic acid, and K_4_[Fe(CN)_6_]·3H_2_O. The presence of sulfonate groups on the catalyst’s surface enhanced its water dispersibility and magnetic recoverability. Similarly, an MCM-supported Fe_3_O_4_-based nanostructured catalyst, Fe_3_O_4_@Fe-Cu/MCM-41, was prepared by Abdollahi-Alibeik et al. for BH C–N cross-coupling reaction [90]. Owing to its magnetic behavior, the catalyst demonstrated excellent recoverability and reusability without significant loss of activity or magnetic properties. 

Very recently, Amali et al. developed a heterogeneous magnetic nanocatalyst by immobilizing Co NPs onto hollow Fe_3_O_4_ nanospheres that are covered with a porous covalent triazine framework, Co/Fe_3_O_4_@triazine [91]. This magnetically separable catalyst obtained from this process demonstrated exceptional performance in ligand-free BH N-arylation reaction. The nitrogen-enriched porosity structure of the nanocatalyst enhanced the ability to interact with active centers, kept the Co nanoparticles apart to preserve their activity, and prevented them from clumping together during reactions. This guaranteed the stability of the catalytic sites, enabling their reuse without experiencing any decrease in activity. Zhang et al. developed an innovative pectin-functionalized magnetic Fe_3_O_4_ nanoparticle incorporating Pd (Fe_3_O_4_@Pectin/Pd) as a heterogeneous nanocatalyst for Suzuki and BH cross-coupling reactions [92]. Owing to its excellent effectiveness, environmental friendliness, and reusability, various biphenyl and arylamine derivatives were efficiently produced. Additionally, the catalyst’s performance was maintained over multiple recycling cycles. Recently, Rouzifar et al. derived an innovative multifunctional catalyst containing Fe-MOF functionalized with a Co complex (Fe-MIL-101-isatin-SB-Co) [93]. This modified MOF serves as an efficient heterogeneous and recyclable catalyst for Ullmann, BH, Hirao, Hiyama, and Mizoroki–Heck cross-coupling reactions involving various aryl halides, phenylboronic acid, and phenyl tosylate with phenols, anilines, heterocyclic amines, triethyl phosphite, tri-ethoxyphenylsilane, and alkenes, yielding the desired coupling products in moderate to high yields. Dubey et al. proposed a Pd-doped Fe_3_O_4_ nanocatalyst with a polydopamine (PDA) coating (Pd/Fe_3_O_4_@PDA) as an efficient catalyst for the Ullmann homocoupling of various aryl halides, arylboronic acids, and aryldiazonium salts in aqueous media with RM-β-CD [94]. The catalyst was magnetically recoverable and reusable for up to five cycles without significant loss of catalytic activity. To the best of our knowledge, this was the first report of a magnetically recoverable catalyst used for Ullmann homocoupling of these substrates in water. Mansoori et al. synthesized a new bis(NHC)-Pd(II) catalyst supported on magnetic Fe_3_O_4_@SBA-15 [95]. The material was further immobilized with trans-[Pd(Cl)_2_(SMe_2_)_2_] complex to form Fe_3_O_4_@SBA-AP-CC-bis(NHC)-Pd(II). This supported Pd(II) complex was reported to efficiently catalyze the direct monoarylation of ammonia with iodo-, bromo-, and chloroarenes with greater selectivity. Similar to the previous reports, the catalyst could be magnetically separable and reusable for several cycles with minimal deactivation. Hemmati et al. reported a novel magnetic Fe-oxide-based nanocatalyst (Fe_3_O_4_@PVA/CuCl) with an Fe_3_O_4_ NP coating of with polyvinyl alcohol (PVA) followed by coordination with CuCl [96]. The reported material exhibited high efficiency in the N-arylation of amines via Ullmann-type coupling reactions, facilitating the formation of C–N bonds between aryl halides and amines. The catalyst proved to be highly stable, maintaining its catalytic performance for at least seven consecutive cycles without significant degradation. Ding et al. reported a non-toxic and cost-effective Fe complex (iron ethylene-1,2-diamine) over carbon nanotubes (CNTs) as a single-atom heterogeneous catalyst (Fe-Nx/CNTs) for C–N bond formation reaction from aromatic amines and ketones [97]. The synthesized catalyst exhibited high efficacy in this process, demonstrating a wide range of substrate variety. Furthermore, the catalyst also demonstrated industrial potential by being reusable for seven cycles without significant activity loss.

In another work, Sun et al. demonstrated a fascinating protocol for the synthesis of single-atom Fe catalyst (Fe_1_-N-C) derived from zeolitic imidazolate frameworks (ZIF), for selective ammoxidation reactions, i.e., the elimination reaction between a benzyl alcohol and aqueous NH_3_ [98]. The reported Fe_1_-N-C catalyst was proven to be very efficient for the synthesis of various nitriles (–CN) from alcohols in water under mild conditions, offering chemo selectivity, recyclability, and high efficiency. Most importantly, this Fe_1_-N-C catalyst was a valuable addition for nitrile synthesis, important in fine chemical industry as well as other research sectors. Another effective method for forming single C–N bonds from stable and easily accessible substrates, such as amines and alcohols, is hydrogen auto transfer amination, often known as borrowing hydrogen [99]. Many believe it to be the most environmentally friendly and atom-efficient way to make complex amines [100].

## 5. Oxidation Reactions

Organic oxidation reactions are considered to be one of the well-studied and significant organic transformation reactions due to their versatile applicability in different sectors like fine chemicals, pharmaceuticals, drug, and dye synthesis [101,102]. These reactions are significant in both industrial and environmental contexts due to their efficiency and sustainability [102]. Typically, Fe catalysts can activate oxidants like hydrogen peroxide (H_2_O_2_), molecular oxygen (O_2_), or organic peroxides to oxidize substrates, including hydrocarbons, alcohols, olefins, and heterocycles [101]. The reactions often proceed under mild conditions, showcasing the high selectivity and yield of the desired product [102]. Advances in this field continue to optimize catalytic performance, enhancing reaction rates, and minimizing by-products.

### 5.1. Advanced Oxidation Process by Fenton’s Reaction

The first thing one needs to counteract an Fe-catalyzed oxidation reaction is the advanced oxidation process (AOP) or Fenton’s process for the degradation of organic pollutants in waste water remediation [103]. Unlike the traditional Fenton reaction, which uses soluble Fe salts, the heterogeneous variant employs solid Fe catalysts, such as Fe oxides or Fe-impregnated materials [104]. In this process, the solid iron catalyst activates H_2_O_2_ to generate hydroxyl radicals (^•^OH), which are highly reactive and can effectively break down a wide range of organic contaminants [105]. The key advantages of the heterogeneous Fe-catalyzed Fenton reaction include easier recovery and reuse of the catalyst, reduced Fe sludge production, and enhanced stability under various environmental conditions [105]. This method is particularly effective in treating wastewater containing persistent organic pollutants, dyes, and pharmaceuticals [105]. Recent developments in this field focus on improving the catalytic activity, stability, and surface properties of the Fe-based materials [105]. Researchers are also exploring various supports, such as activated carbon, silica, and clays, to enhance the dispersion and accessibility of the Fe active sites [106]. These advancements aim to increase the efficiency and cost-effectiveness of the heterogeneous Fenton reaction, making it a promising solution for large-scale environmental remediation efforts [106]. Recently, Peng et al. created a single Fe-atom N-doped carbon matrix (Fe-N-C) as a superior heterogeneous catalyst for the activation of peroxymonosulphate (PMS) to degrade dissolved pollutants like paracetamol, ciprofloxacin, Bisphenol-A, etc., from waste water [107]. Interestingly, the N-doped carbon cluster was derived from pyrolysis of a sea water pollutant, namely ‘Enteromorpha’ at 900 °C. Radical quenching and electrochemical investigation confirm high-valence Fe-oxo species and an electron-transfer pathway for nonradical oxidation. Another study explaining the activation of PMS was also reported by Madihi-Bidgoli et al., in which a carbon nanotube (CNT)-encapsulated Fe_2_O_3_ nanocatalyst was used for photocatalytic azurobine degradation under UVA-LED irradiation [108]. The azurobine degradation was found to be more accelerated by sulphate radicals than ^•^OH radicals. The recyclability of the material was also analyzed and the response was found to be positive with a good number of catalytic cycles. Chen et al. designed a CNT-supported heterogeneous Fenton-like system, Fe-OCNT, for selective oxidation of methylene blue (MB) and chrysoidine G [109]. The catalyst, Fe-OCNT, fabricated by attaching Fe ions to the surface of oxidized CNT, was allowed to activate H_2_O_2_, and then the Fenton’s process was followed. This selective oxidation is attributed to ^•^OH radicals adsorbed on the CNT surface. This selective oxidation system is found to be effective across a broad pH range (4.0 to 9.0), with excellent resistance to traditional ^•^OH radical quenching agents.

**Scheme 7:**
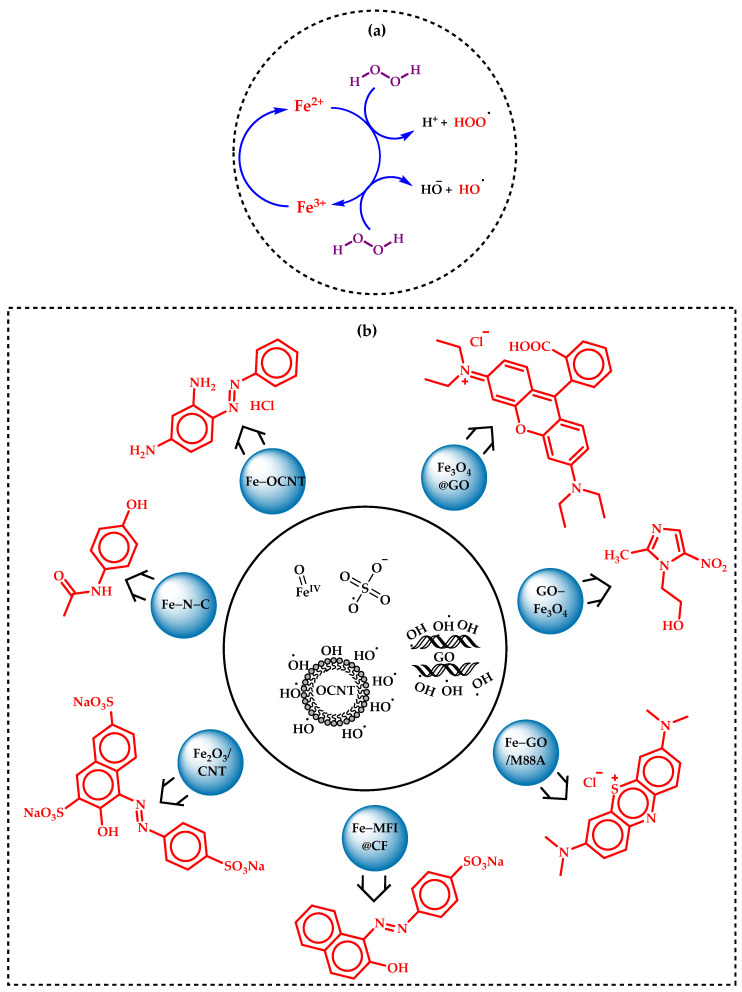
(**a**) Classical pathway of Fenton’s process, (**b**) AOP performed by some reported heterogeneous Fe catalysts (blue) along with the degradative pollutants (red). The inset circle represents the active species of AOP.

Xie et al. demonstrated a graphene oxide (GO)-supported heterogeneous Fe-MOF (Fe-GO/M88A) for a photocatalytic Fenton reaction aimed at water purification [110]. This MOF exhibited significantly improved the catalytic efficiency of MB separation from water samples. The GO/M88A catalyst was effectively treated with urban textile wastewaters and demonstrated high degradation efficiency for MB (98.81%) and bisphenol-A (97.27%) as a catalyst in a photo-Fenton degradation process. These findings highlight the potentiality of the GO/M88A membrane for water purification and environmental protection applications due to its excellent separation performance, stability, and photo-Fenton activity. Dye-polluted wastewater was treated via AOP by Pervez et al. using Fe_3_O_4_-impregnated GO system (Fe_3_O_4_@GO) followed by activation with persulfate (K_2_S_2_O_8_) [111]. This method efficiently degraded Rhodamine B (RhB) to 95% from 25% with Fe_3_O_4_ alone. Nevertheless, after repeated usage, the system retained its true catalytic activity as well as low Fe leaching. Considering the degradation mechanism, rather than the ^•^OH radicals the surface-bound SO^4−^ was found to be the active species for RhB breakdown. Görmez et al. demonstrated an electro-Fenton process using a GO immobilized Fe_3_O_4_ catalyst for the mineralization and breakdown of chloramphenicol and metronidazole antibiotics [112]. The mineralization rate for chloramphenicol was reported to be 73% and that for metronidazole was 86%. Remarkably, over ninety-nine percent of these antibiotics were found to be degraded using this GO-Fe_3_O_4_ catalyst. Afterwards, the durability of the catalyst in these hazardous conditions was shown by its capacity to maintain its heterogeneity for minimum of four consecutive electro-Fenton cycles. Again, Le et al. synthesized an Fe-rich MFI-zeolite-modified carbon felt (CF) material, Fe-MFI@CF, as a heterogeneous catalyst in an electro-Fenton (EF) process for the degradation of acid orange 7 (AO7) [113]. The MFI@CF catalyst was reported to catalyze the full degradation of AO7 (0.1 mM) in 40 min. However, the system exhibited encouraging efficacy, achieving a 26.6% reduction in total organic carbon (TOC) after 8 h at a pH level of 6.5. Scheme 7 depicts some significant Fe-based heterostructures for AOP in waste water remediation.

### 5.2. Oxidation of Benzyl Alcohols

Selective oxidation of benzyl alcohols (BAs) to benzaldehydes (BALs) is a crucial transformation in organic chemistry, often used to synthesize fine chemicals and pharmaceuticals [114]. Benzaldehyde is a highly significant and versatile organic compound that has extensive utility in various industrial applications [115]. This process typically involves using various catalysts, including metal-based, metal-free, and heterogeneous catalysts, to achieve high selectivity and efficiency [116,117,118]. Fe-catalyzed selective oxidation of BAs to BALs is an efficient and environmentally friendly process [119]. Fe-based catalysts facilitate the oxidation reaction under mild conditions, often using O_2_ or H_2_O_2_ as the oxidant. This method provides a high selectivity for BAL with minimal over-oxidation to benzoic acid. The process is notable for its use of readily available and non-toxic Fe catalysts, making it a sustainable choice for industrial applications. Additionally, these catalysts can often be reused without significant loss of activity, enhancing the overall efficiency and cost-effectiveness of the oxidation process. Lots of reports are available in literature for the Fe-catalyzed selective oxidation of BAs to BALs. Crombie et al. synthesized a heterogeneous catalyst of Pd-Fe alloys supported on titania (TiO_2_) as an efficient catalyst in the specific oxidation of BA to BAL without any external oxidizing agent [118]. This mechanism of the studied reaction depends on the in situ generation of H_2_O_2_ from molecular hydrogen (H_2_) and O_2_, and interestingly, the reaction did not take place with molecular O_2_ alone. The oxidation rate of BA was found to be much greater when utilizing supported Pd-Fe NPs as compared to Pd-Au or Pd-only counterparts. A mild method for fabricating spherical NiFe_2_O_4_ NPs as a catalyst for selectively oxidizing BA to BAL was presented by Iraqui et al. [120]. This nanocatalyst displayed a high oxidation ability with 85% conversion of BA and 100% selectivity using tert-butyl hydroperoxide (TBHP) at 60 °C in 3 h. Owing to its magnetic behavior, the NiFe_2_O_4_ nanocatalyst was easily separable and reusable for up to five consecutive cycles without hampering catalytic activity. The comparative rates of oxidation of BAs to BALs in O_2_ and H_2_O_2_ environments were investigated by Baruah et al. over an Fe_2_O_3_ photocatalyst embedded in halloysite nanotubes (HNTs) [121]. The catalysis results demonstrated that the Fe_2_O_3_-HNT nanocatalyst achieved greater activity in the presence of the H_2_O_2_ oxidant than O_2_. The mechanism of oxidation was also investigated by electron spin resonance (ESR) and Raman spectroscopic analysis, which provided direct evidence of the involvement of superoxide radical bound Fe (III) species in the BA oxidation reaction. Owing to its suitable band gap (∼2.14 eV), the catalyst enabled absorption under visible light irradiation. A new synthesis method was represented by Wei et al. for the preparation of a Mott–Schottky type N-doped carbon-supported single-atom Fe (SA-Fe/Nx-C) catalyst for the selective oxidation of BA to BAL with superior catalytic ability [122]. The N source of the catalyst was appeared from carbon nitride which also acted as a templating agent during the catalyst synthesis. The high catalytic activity was attributed to the tetra-coordinated FeN_4_ structure of the material, which facilitates electron transfer from metallic Fe to N atoms at the Mott–Schottky interface. Furthermore, the systematic introduction of N into carbon through the precise manipulation of the carbon nitride ratio during catalyst synthesis facilitated the modulation of electron transfer in metallic Fe. Consequently, this approach significantly improved the selectivity of BA oxidation. In the meantime, the core–shell nanocatalysts gained significant importance in the catalysis sector due to their unique properties and diverse applications in different areas [123,124]. These structures consist of a core material surrounded by a shell of another material, offering synergistic effects and enhanced functionalities compared to their individual components [123,125]. Recently, Xu et al. introduced Fe_3_O_4_-based core–shell nanocatalysts (Fe_3_O_4_@Cu_2_O) and Fe_3_O_4_@Cu_2_O–Cu for the aerobic oxidation of BAs to BALs, employing 2,2,6,6-tetramethylpiperidine-N-oxyl (TEMPO) and N-methylimidazole (NMI) as co-catalysts [126]. When combined with both TEMPO and NMI, these materials exhibited excellent catalytic activities in the aerobic oxidation of BAs under ambient conditions. The materials demonstrated recyclability and robustness, maintaining activity with less than a 10% drop in performance over seven consecutive runs. Their magnetic properties of these materials facilitated the easy separation after the reaction using an external magnet. The facile synthesis and catalytic performance of a newly synthesized core–shell α-Fe_2_O_3_@Au nanocomposite were emphasized by Paul et al. [127]. The reported results evident for the excellent catalytic performance of the α-Fe_2_O_3_@Au in BA oxidation, with up to five repeated catalytic cycles. Moreover, Zheng et al. derived a magnetically retrievable core–shell photocatalyst of Fe_3_O_4_, cadmium sulfide (CdS), and carbon quantum dots (CQDs), Fe_3_O_4_@CdS@CQDs, for the selective oxidation of BA with in situ generated H_2_O_2_ [128]. Additionally, novel Z-scheme photocatalysis was found to follow the mechanistic route of the oxidation process. The outcomes of BA oxidation by recently reported Fe heterostructures are summarized in Table 1.

**Table 1:** Significant Fe-based heterostructures recently reported for BA oxidation reaction.

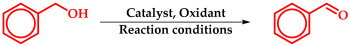						
Entry	Catalyst	Oxidant	% Conv. (BA)	% Sel. (BAL)	% Yield (BAL)	Ref.
1	Pd-Fe@TiO_2_	H_2_O_2_	97	100	−	[118]
2	NiFe_2_O_4_ NPs	TBHP	85	100	−	[120]
3	Fe_2_O_3_-HNT	O_2_, H_2_O_2_	88, 92	100, 100	−	[121]
4	SA-Fe/Nx-C	O_2_	92	97	−	[122]
5	Fe_3_O_4_@Cu_2_O	air	−	99	99	[126]
6	α-Fe_2_O_3_@Au	air	−	98	96	[127]

% Conv. (% conversion), % Sel. (% selectivity), and the reaction conditions are as per the respective article.

**Table 2:** Olefin epoxidation results of reported Fe-N/C and Fe/PMA@CIN-1 catalysts [129,130].

Catalyst	Oxidant	Reactant	Product(s)	% Conv. ^[a]^	% Yield	% Sel. ^[b]^
Fe–N/C	O_2_ (1 atm)	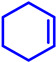	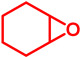	99	91	91
		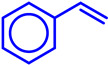	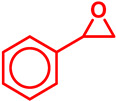	70	17	12
		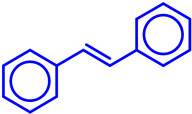	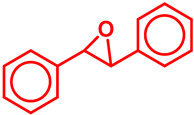	78	67	52
		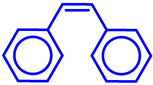	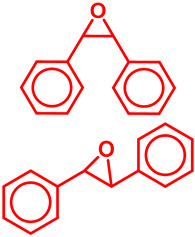	79	96	76
Fe/PMA @CIN-1	H_2_O_2_ (1 mmol)	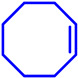	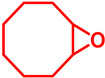	88	−	99
		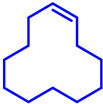	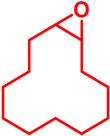	58	−	99

Solvent: acetonitrile (CH_3_CN), Time: 6–17 h (Fe-N/C) and 8 h (Fe/PMA@CIN-1), Temperature: Room temperature (Fe-N/C) and 80 °C (Fe/PMA@CIN-1), % Conv. ^[a]^ = % conversion of the olefin, % Sel. ^[b]^ = % selectivity of the epoxide.

### 5.3. Epoxidation Reactions

The epoxidation reaction offers a sustainable and efficient method for synthesizing epoxides, important intermediates in organic synthesis [131]. Epoxides are highly valuable intermediates and building blocks in the synthesis of various chemicals, including pharmaceuticals, agrochemicals, and fine chemicals [132]. Epoxidation can be achieved through different methods, such as using peracids, peroxyacids, or metal-catalyzed processes [133]. Among these, metal-catalyzed epoxidation reactions, particularly those involving transition metals like Ti, Co, Cu, and Fe, have gained significant attention due to their efficiency, selectivity, and compatibility with diverse functional groups [134,135,136]. These reactions play a crucial role in the synthesis of complex molecules and are essential tools in the repertoire of synthetic chemists [136]. Malko et al. synthesized a heterogeneous Fe-incorporated carbon-supported catalyst for effective epoxidation of cyclic olefins (like cyclohexene) in O_2_ environment [129]. This recyclable catalyst was reported to successfully produce the desired epoxidation products with a very high % yield (Table 2). Yu et al. developed an Fe and phosphomolybdic acid (PMA) immobilized covalent organic framework (COF), Fe/PMA@COF, for the selective and efficient epoxidation of cyclooctene with H_2_O_2_ (Table 2) [130]. This report on the Fe-immobilized COF presented an extraordinary heterogeneous catalyst that efficiently oxidized olefins in an inexpensive way, and displayed the magnificent properties of the COF as the catalytic support for such reactions. In the field of chemical industry, another significant epoxidation reaction is transition-metal-catalyzed styrene epoxidation, because the resulting product, styrene oxide, serves as a key building block for the production of various invaluable chemicals, including plastics, pharmaceuticals, and fine chemicals [137,138]. Epoxidized styrene derivatives also find applications in polymerization reactions to produce polystyrene and as intermediates in the synthesis of surfactants, detergents, and epoxy resins [139,140]. Recently, Yang et al. synthesized an amorphous alumina (Al_2_O_3_)-supported FeO_x_ heterogeneous catalyst for the selective conversion of styrene-to-styrene oxide with a high catalytic efficiency [137]. Fe atoms were regarded to enhance the adsorption of styrene onto the catalytic active sites of the clusters, paving the way for epoxidation. Additionally, the defective structure of the amorphous Al_2_O_3_ nanosheet also triggered the reactivity of the catalyst by preventing the FeO_x_ moieties from agglomeration during the epoxidation process. Recently, a magnetic Fe_3_O_4_ core and a porous CuSiO_3_ combined heterogeneous core–shell nanocatalyst was designed by Wu et al. for a highly selective styrene epoxidation reaction [141]. The catalyst outperformed pure Fe_3_O_4_, pure CuSiO_3_, or a physical combination of the two in styrene epoxidation with TBHP. Because of its great stability and ease of recycling, it maintained its catalytic activity and structural integrity even after six uses. Similarly, Liu et al. developed a heterogeneous spinal cobalt ferrate nanocatalyst, CoFe_2_O_4_, for styrene epoxidation with TBHP as the oxidant [142]. The activity of this spinal on epoxide generation was reported to significantly outperform its monometallic equivalents i.e., Co_3_O_4_ flakes and Fe_2_O_3_ rods. Due to its large surface area, mesoporous structure, and abundance of surface metal redox couples resulting from its bimetallic structure, the CoFe_2_O_4_ produced a remarkable catalytic activity. However, the material retained its true catalytic ability as well as heterogeneity for up to five consecutive runs.

### 5.4. Hydroxylation Reactions

Hydroxylation reactions are crucial in both biological and synthetic chemistry, playing a key role in the metabolism of various substances and the synthesis of complex molecules [143,144]. In biological systems, hydroxylation is often mediated by enzymes such as cytochrome P450 monooxygenases, which facilitate the oxidation of organic substrates [145]. In industrial and laboratory settings, hydroxylation can be achieved using a variety of catalysts and oxidizing agents, enabling the production of valuable chemicals, pharmaceuticals, and intermediates for further chemical transformations [146,147,148,149]. Considering Fe-catalyzed hydroxylation reactions, Salazar-Aguilar et al. derived an Fe-based MOF, Fe-BTC for the direct hydroxylation of phenol to synthesize dihydroxy benzene in the presence of H_2_O_2_ [150]. The active species of the hydroxylation to occur was reported as ^•^OH species from H_2_O_2_ breakdown. Very recently, a recyclable nanocatalyst for benzene hydroxylation was created by Wu et al. using cupric acetylacetonate grafted Fe-doped mesoporous silica (Cu/Fe-SBA-15) with extensively scattered metallic sites [151]. Owing to the synergistic effect of the doped Fe sites and the grafted Cu sites, the synthesized Cu/Fe-SBA-15 catalyst showed outstanding catalytic activity in the direct hydroxylation of benzene with H_2_O_2_ as the oxidant. Yue et al. also designed an MFI-zeolite-supported Fe-based catalyst (Fe-MFI zeolite) for the H_2_O_2_ mediated hydroxylation of benzene [152]. Compared to the pure silica zeolite seed sol, Fe-MFI zeolite exhibits a higher conversion of 12.1% of benzene with a selectivity of 97% at 60 °C during the reaction in H_2_O_2_ environment. Focusing on the effects of the ion-exchange sequence on the physicochemical and catalytic characteristics, Xiao et al. investigated an Fe, Cu-based bimetallic catalyst over zeolite support, Fe-Cu/beta zeolite [153]. When subjected to benzene hydroxylation with H_2_O_2_, the catalyst displayed outstanding catalytic activity with a ~43% conversion of benzene with ~96% product selectivity. Another efficient Fe-based catalyst for benzene hydroxylation was reported by Lu et al. through covering Fe NPs with a layer of N-doped carbon (Fe@NC) [154]. The carbon shells prevented Fe leaching and enhance selective benzene adsorption with their hydrophobic surface. However, Fe@NC displayed very good selectivity and long-lasting catalytic efficacy of benzene hydroxylation with 16% yield of phenol and 95% selectivity. Similarly, another core–shell carbon encapsulated Co-Fe nanostructure was designed by Zeng et al. for the highly selective hydroxylation of phenolic compounds [155]. The catalyst achieved higher results, with 27% phenol with 97% selectivity. The selective hydroxylation of benzene to phenol by some interesting Fe-based catalysts is depicted in Table 3. 

### 5.5. Oxidative Coupling Reactions

An oxidative coupling reaction is a chemical process in which two molecules or molecular fragments are joined together with the concurrent oxidation of a substrate [156,157]. The process can be catalyzed by various metals, including iron, copper, and palladium, which facilitate the removal of electrons from the substrates, promoting their coupling [158]. Oxidative coupling is widely used in organic synthesis to construct complex molecular structures efficiently and selectively [158]. One of the oxidative coupling reactions that has received a lot of attention is the selective production of BINOL from 2-naphthol [159]. According to the literature, this coupling reaction may be facilitated by a variety of metal catalysts, including salts, metal Schiff-base complexes, metal NPs supported on inorganic mantles, and metal oxide nano catalysts [160,161]. Out of the various transition metal catalysts, Fe-based catalysts are known to be most effective for such conversion [162,163]. The majority of the studies focus on using O_2_ as an oxidant, whereas there are only a countable number of articles that address the selective oxidation of 2-naphthol when H_2_O_2_ is present. When 2-naphthol is oxidized with H_2_O_2_, it produces 1,2-dihydroxynaphthalene and 1,2-naphthoquinone as by-products; however, the yield of BINOL is very low. In the presence of O_2_, the observed reaction time is considerably longer because O_2_ is a mild oxidant. Although the oxidant H_2_O_2_ (30% *w*/*v*) is effective in converting a significant portion of the reactant quickly, it often lacks control over selectivity during the reaction. Interestingly, Baruah et al. also apply the HNT-embedded Fe(III) catalyst (Fe_2_O_3_-HNT) for the oxidative coupling of 2-naphthol to synthesized selective BINOL [121]. On the other hand, enantioselective oxidation of 2-naphthol has gained a lot of interest due to the versatilities of chiral (R/S) BINOL derivatives in different areas. Interestingly, Horibe et al. developed chiral Fe(II)-di-phosphine oxide complexes for the selective synthesis of chiral BINOL via oxidative coupling reactions [163]. This chiral catalyst exhibited excellent activity of R-BINOL production up to 98% yield with 68% enantiomeric excess (ee). Similarly, Wu et al. also demonstrated an efficient protocol of the synthesis of chiral BINOL through an in situ generated Fe catalyst by enantioselective oxidative coupling of 2-naphthol [162]. The catalyst was synthesized by simultaneous addition of Iron(II) perchlorate hydrate and a bis-quinolyl diamine ligand [(1*R*,2*R*)-*N*^1^,*N*^2^-di(quinolin-8-yl) cyclohexane-1,2-diamine along with the 2-naphthol reaction mixture. Different 2-naphthol derivatives were also analyzed by this method and it was noted that moderate to good outcomes were attained with the formation of respective R-BINOL derivatives in terms of % yield and ee. However, to the best of our knowledge, Fe-based heterogeneous chiral catalysts have not been widely used for such enantioselective oxidation reactions. Table 4 represents some recently reported Fe-based catalysts for the oxidative coupling of 2-naphthol to BINOL.

## 6. Epoxide Ring Opening Reactions

**Scheme 8:**
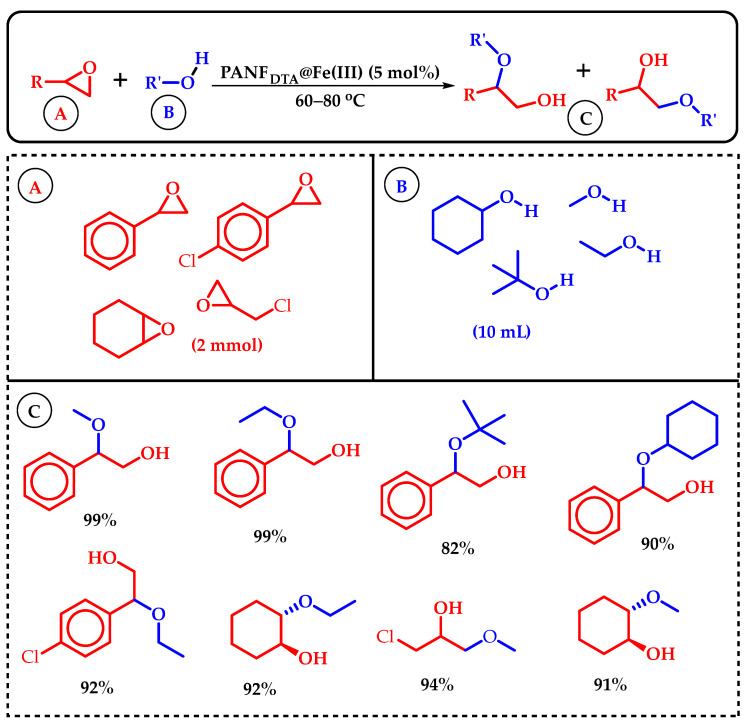
Ring opening of epoxides catalyzed by PANF_DTA_@Fe(III) reported by Shi et al. [175]. In the scheme, A and B are the reactants, and C is the product. The % yields of Cs are mentioned with the respective structure.

Epoxide ring opening is a fundamental organic reaction catalyzed by acids, bases, or nucleophiles, wherein an epoxide molecule undergoes cleavage to form a diol or other functionalized products [164,165,166]. Mechanistically, the reaction involves the attack of a nucleophile at the electrophilic carbon of the epoxide ring, leading to the formation of a new bond and opening of the ring [167,168]. Epoxide ring opening reactions are widely employed in organic synthesis to construct complex molecules and functionalize organic compounds with diverse applications in pharmaceuticals, materials science, and fine chemicals [169,170,171]. Typically, transition metals such as Ti, Al, Zn, and Fe serve as catalysts [169,170,171,172,173]. Nagarjun et al. synthesized an Fe-based MOF, MIL-101(Fe), as a highly active, regioselective heterogeneous Lewis-acid catalyst for ring-opening styrene oxide [174]. The MIL-101(Fe) exhibited superior catalytic activity of styrene epoxide ring opening by indole and its derivatives as a nucleophile with a 70–90% yield of the desired product. Furthermore, the heterogeneity of the catalyst was confirmed through leaching tests, and its activity was maintained over four catalytic cycles. Shi et al. derived a polyacrylonitrile fiber (PAN F_DTA_) supported Fe(III) heterogeneous catalyst for epoxide ring opening reaction [175]. In presence of the catalyst, the reaction proceeded with methanol as the nucleophile and demonstrated ~80–99% yield of the open-chain ꞵ-methoxy alcohol product (Scheme 8). In addition, the catalyst maintained its properties during long-term storage without additional protection, and there was no discernible decline in the catalytic activity for recycling over 20 cycles. Wang et al. synthesized molecular sieve (PKU-1) supported Fe catalyst, Fe-PKU-1 for ring opening of cyclohexanone using water as the nucleophilic agent [176]. It was reported that both the SN^1^ and SN^2^ pathways are favored by the reaction for the selective production of *trans*-1,2-cyclohexanediol over the *cis*-form with excellent yield. Moreover, the Fe-PKU-1 is reported to be stable for up to five catalytic cycles without hampering the % yield as well as the selectivity of the *trans*-diol.

## 7. Future Perspectives

In the realm of organic synthesis, the future of heterogeneous Fe catalysis holds promising prospects. With ongoing advancements in catalytic methodologies, Fe-based catalysts are poised to play a pivotal role in sustainable and environmentally friendly organic transformations. Leveraging the abundance, low cost, and eco-friendliness of iron, researchers are exploring innovative approaches to enhance the efficiency, selectivity, and scope of Fe-catalyzed reactions. Moving forward, one can anticipate the development of novel Fe-based catalysts with tailored structures and compositions, aimed at addressing specific challenges in organic synthesis. This includes the design of heterogeneous catalysts with well-defined active sites, improved stability, and enhanced catalytic performance under mild reaction conditions. Moreover, the integration of Fe catalysis with emerging technologies, such as flow chemistry and photochemistry, offers exciting avenues for efficient and selective organic transformations. Harnessing the synergistic effects of Fe catalysis with these advanced techniques can enable the development of streamlined processes with reduced energy consumption and waste generation. Furthermore, exploring the catalytic potential of Fe-based materials in tandem with sustainable reaction media and renewable feedstocks holds promise for greener and more sustainable chemical synthesis. The integration of Fe catalysis with biocatalysis and cascade reactions could lead to the development of efficient and environmentally benign synthetic routes for the production of valuable organic molecules. Briefly, the future of heterogeneous Fe catalysis in organic synthesis is characterized by innovation, sustainability, and versatility. By harnessing the unique properties of iron and leveraging advancements in catalytic design and methodologies, Fe-based catalysts are poised to make significant contributions to the development of efficient and sustainable synthetic routes for diverse organic transformations.

## 8. Conclusions

The application of heterogeneous Fe catalysis in these diverse transformations underscores its versatility and potential as a sustainable and cost-effective alternative to precious metal catalysts. By harnessing the unique reactivity of Fe, chemists have been able to develop innovative synthetic methodologies that are not only efficient and selective but also environmentally benign. Moreover, the abundance and low cost of Fe make these catalysts accessible to a wide range of researchers, both in academia and industry, facilitating the further exploration and development of heterogeneous Fe-based catalytic systems.

## Figures and Tables

**Table 3:** Selective hydroxylation of benzene to phenol by some reported Fe-based heterostructures [149,151,152,153,154].

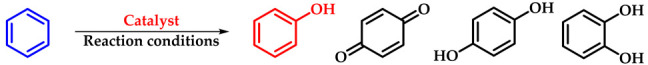					
Entry	Catalyst	Amount (mg)	Temp. (°C)	Time (h)	% Sel. (Phe.)
1	Fe-MFI-Zeolite	100	60	2	97
2	Fe-Cu/Beta Zeolite	50	60	6	96
3	Cu-Fe/SBA-15	30	60	3	93
4	Fe-NC	30	60	4	95
5	CuO-Fe_2_O_3_/Fe_3_O_4_	50	40	24	85

All the reactions were performed in CH_3_CN solvent and H_2_O_2_ as oxidant, Temp. = Temperature, % Sel. (Phe.) means % Selectivity of phenol.

**Table 4:** Results of oxidative coupling of 2-naphthol to BINOL by some recently reported Fe catalysts.

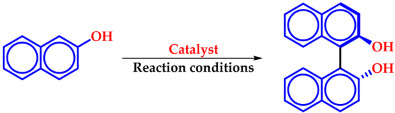								
Entry	Catalyst	Amount	Oxidant	Temp.	Time (h)	% Yield ^[a]^	% ee ^[b]^	Subs. ^[c]^
1	Fe_2_O_3_-HNT	30 mg	O_2_, H_2_O_2_	RT	2, 1	89, 72	−	1
2	Fe(OTf)_2_-ligand	5 mol%	TBHP	−20–10 °C	60	77–98	6–70	19
3	Fe(ClO)_4_-ligand	10 mol%	O_2_	50 °C	5–18	51–99	12–62	12
4	[Fe(salan)-ν-(OH)]_2_	4 mM	O_2_	60 °C	72	87	−	1

The reactions were performed in CH_3_CN solvent, except for [Fe(salen)-n-(OH)]_2_ which was performed in toluene, RT = Room Temperature, % Yield ^[a]^ = % Yield of BINOL, % ee ^[b]^ = % enantiomeric excess of R–BINOL, Subs. ^[c]^ = substrate diversity.

## Data Availability

No new data were created or analyzed in this study.
